# Pandora’s Box: A spatiotemporal assessment of elephant-train casualties in Assam, India

**DOI:** 10.1371/journal.pone.0271416

**Published:** 2022-07-13

**Authors:** Rekib Ahmed, Anup Saikia

**Affiliations:** Department of Geography, Gauhati University, Guwahati, India; Texas State University, UNITED STATES

## Abstract

Railways are an indispensable component of sustainable transportation systems, but also exact a toll on wildlife. Wild Asian elephants are often killed by trains in Assam, India, where we assess temporal variations in the occurrences of elephant-train collisions (ETCs) and casualties during 1990–2018. This study also assesses spatially varying relationships between elephant-train collision (ETC) rates and elephant and train densities in the adjoining 10 km^2^ grid cells of 11 prioritized railroad segments using ordinary least squares (OLS) and geographically weighted regression (GWR) models. The temporal analysis indicated that ETCs spiked at certain hours and months. The adult and calf elephant casualties on the railroads were found to be two to fivefold high during the post monsoon season compared to other seasons. During the operation period of meter gauge railroads (1990–1997), the proportions of ETCs and casualties were only 15.6% and 8.7% respectively. However, these increased substantially to 84.4% and 91.3% respectively during the operation of broad gauge railroads (1998–2018). The OLS model indicated that both elephant and train densities explained 37% of the variance of ETC rate, while GWR model showed 83% of the variance of ETC rate. The local coefficient values of GWR indicated that both the predictor variables interplayed significantly and positively to determine ETC rates in the Mariani-Nakachari and Khatkhati-Dimapur railroad segments. However, the relationship between ETC rate and elephant density is significantly negative in the Habaipur-Diphu railroad, implying that the elephant population along this railroad stretch is significantly affected by railways through large scale ETCs. Hence, there is an urgent need to address long-term mitigation strategies so that elephants can be conserved by providing safe passages and survival resources along railway lines.

## 1. Introduction

Although railways offer a sustainable transport mode in terms of energy usage and greenhouse gas emission [1], they slice nature into pieces that often opens a Pandora’s Box of ecological stresses, such as habitat loss and fragmentation [2], barrier effects [3] and wildlife-train collisions (WTCs) [4]. The latter continues unabated, killing countless wildlife species each year across the world [4, 5]. Wild Asian elephants possessing large movement ranges are often killed by trains in the Indian provinces of Assam [6], northern West Bengal [7] and Uttarakhand [8]. In these regions, over 200 railway-induced elephant mortalities were recorded from 1987 to 2015 [9]. India recorded the world’s largest number of elephant–train accidents [10]. Such accidents are the largest source of elephant mortality in India after deaths by electrocution [11–13]. Generally, elephants function as ecosystem engineers and seed dispersers and their substantial mortalities can cause cascading effects on ecological processes [14].

WTCs are often conceived of as a relational effect of changing densities of animal population and traffic volumes [5, 15, 16], although such effects remain unknown in case of elephant-train collisions (ETCs). Spatial patterns of animal density were one of the key indicators of WTCs in Norway [17], Sweden [18] and Poland [19]. It was observed that animal species with low population densities and large movement ranges were highly vulnerable to traffic-induced collisions [20]. On the other hand, increasing train densities in the wildlife ranges not only affects the behaviours of numerous wild animals [15, 21, 22], but also emerged as a conspicuous factor of the WTCs [23]. A positive association often seems to exist between train densities and the quantity of mortalities of large animals from train collisions [24–26]. It is estimated that moose populations declined by 70% due to wildlife-train collision in parts of Alaska [27]. Linear infrastructures increase wildlife-vehicle and -train collisions [28, 29] and pose a grave threat to diverse species including the iconic Asian elephant [4, 30]. Limited studies on ETCs exist [8, 9, 31, 32] and assess land cover changes and habitat fragmentation in affected areas. Biodiversity conservation and environmental costs have to be woven into planning roads and railways [29] and it is clear that ETCs and WTCs are poised to mount as linear infrastructures expand.

ETCs may be a function of two variables: elephant and train densities. Asian elephants require large intact forest patches of upto 440,000 ha for 500 breeding individuals [33] and are forced to cross railway tracks. The frequency and intensity of trains operating in areas with elephant presence, or in close proximity to elephant habitat, is the second variable. Elephant density patterns are often associated with landscape heterogeneity [34], vegetation phenology [35], surface water availability [36] and precipitation variability [37] and determine the behavioural responses and movement intensity of elephants on roads and railways [38–40]. Declining fodder in their fragmented habitats compels elephants to frequently raid crops. Additionally, elephants are also attracted by the smell of country liquor made by villagers in rural areas of India and Nepal [41, 42]. Elephants are also known to enter urban areas on the fringes of protected areas. Such behaviour leads to human elephant conflict (HEC) and the costs to both elephants and humans can be immense. In Assam’s Sonitpur district, 22 elephants were killed by poisoning waterholes in 2001 [43, 44] following crop raiding incidents that devastated rural livelihoods and savings. In 2019, in the army cantonment in Narangi, Guwahati on the fringes of the Amchang Wildlife Sanctuary, iron spikes were laid on the road to prevent elephants from entering the premises, causing the deaths of two wild elephants by septicemia [45]. Entering anthropogenic landscapes necessitates elephants to cross roads and railway tracks. The behavioural responses of elephants to railway traffic can be classified as non-responder, avoider and speeder [46]. A non-responder fails to or is unable to respond to moving vehicles either because it cannot detect it in time or because it does not perceive the oncoming vehicle as a threat since they lack predators similar to the oncoming train (or any other vehicle) [46]. A speeding train approaching on a curved railroad track at night or in the wee hours can give elephants very little reaction time. An avoider, as the term suggests, is an animal that avoids crossing the track for certain reasons, but this behaviour is not always possible when the railway tracks cause habitat fragmentation or slice into a forest stretch. Speeders are animals that tend to “flee as a primary response to threat” [46], although their escape response may not necessarily be successful. Non-responder individuals have a greater elephant-train collision (ETC) risk, since they do not perceive an approaching train as a threat to their lives. Moreover, spatially increased train densities make it more difficult for numerous animals to assess the distance of approaching trains and, consequently, they become trapped on the railroads due to decreasing time interval between two successive trains [5]. Compared to vehicles, train density is much lower on railroads that facilitate higher speed train flows and larger volumes of goods and people [47], the substantial train-spilled food that is generated attracts wild animals [48, 49].

WTC related literature indicates that there are a few studies that apply statistical models to link the spatial associations between WTC incidence and various spatial characteristics such as landscape configuration and railway tracks. Such studies are mainly based on global regression models, such as logistic regression [7, 50] and generalized linear model [51], due to their potency for determining global associations between response and predictor variables. However, global regression models often violate the basic assumptions of residuals’ independence (no spatial autocorrelation) and residuals’ constant variance (homoscedasticity) in two specific situations. The first is when the values of a bio-geographic variable are spatially and significantly correlated in nearby locations, termed spatial autocorrelation [52]. The second is when the spatial relationships between some bio-geographic variables significantly differ from one place to another, also known as spatial non-stationary [53]. In the latter instance, global regression models generally exhibit a consistent spatial relationship and ignore the potential variations of spatial relationships between variables.

Geographically weighted regression (GWR) is an approach that has received considerable attention, when using spatial autocorrelation to represent spatially varying relationships [54]. GWR is capable of capturing locally varying associations by permitting regression parameters to vary over localities. The local parameter estimates of the GWR model are based on a distance weighting scheme, implying that neighbouring observations have more influence in the estimation than the distant observations. GWR generates many local regression statistics including local residuals, coefficient of determination (R^2^) and coefficients for each sampling point, while it also produces global R^2^ values, and corrected Akaike Information Criterion (AIC_c_). Hence, this model could be a convenient tool for assessing spatially varying relationships between ETCs and elephant and train densities. Identifying such a relationship could help develop local strategies to minimize ETCs. Besides using GWR, the present study also uses ordinary least squares (OLS) regression, which is a classical form of global regression models, to differentiate the model performances between OLS and GWR. Since ETCs vary during the hours of the day, seasonally and over the years, we looked into these temporal aspects as well. In particular, we distinguish between late night and early morning hours, seasons linked with the harvesting of crops like paddy and maize and years disaggregated on the basis of the width of railway tracks. The main objectives of the present study were: (i) to assess temporal variations of ETCs and casualties in Assam for the period 1990–2018; and (ii) to assess the spatially varying relationship between ETCs and elephant and train densities in grid cells of certain prioritized railroad segments. ETCs and casualties differ in that a single ETC may cause one or multiple elephant mortalities and 5 to 6 elephants succumbing to a single ETC is not unknown [10]. Throughout this analyses, we refer to elephant, and not human, casualties.

## 2. Materials and methods

### 2.1 Study area

Forming a part of the Indian zoo-geographic sub-region and railway-based economy, Assam, a landlocked state in north-east India, is of exceptional railway-ecological interest. It extends latitudinally from 24˚3′ N to 28˚2′ N and longitudinally from 89˚4′ E to 97˚1′ E. The total route length of the Assam railways in 1988–89 was 2338 km and these entire stretches were characterized by meter gauge railroads (MGR) [55]. To strengthen operational efficiency of these railroads, most of the tracks, barring the Rangiya-Murkongselek and Lumding-Silchar railway sections, were upgraded from MGR (width = 1000 mm) to broad gauge railroad (BGR) (width = 1676 mm) in 1997 [56]. With the completion of gauge conversion project of Rangiya-Murkongselek and Lumding-Silchar section in 2015, the gauge unification process in the state was complete [57] and the total running track length of BGR during 2017–2018 was 2577 km [58].

**Fig 1:**
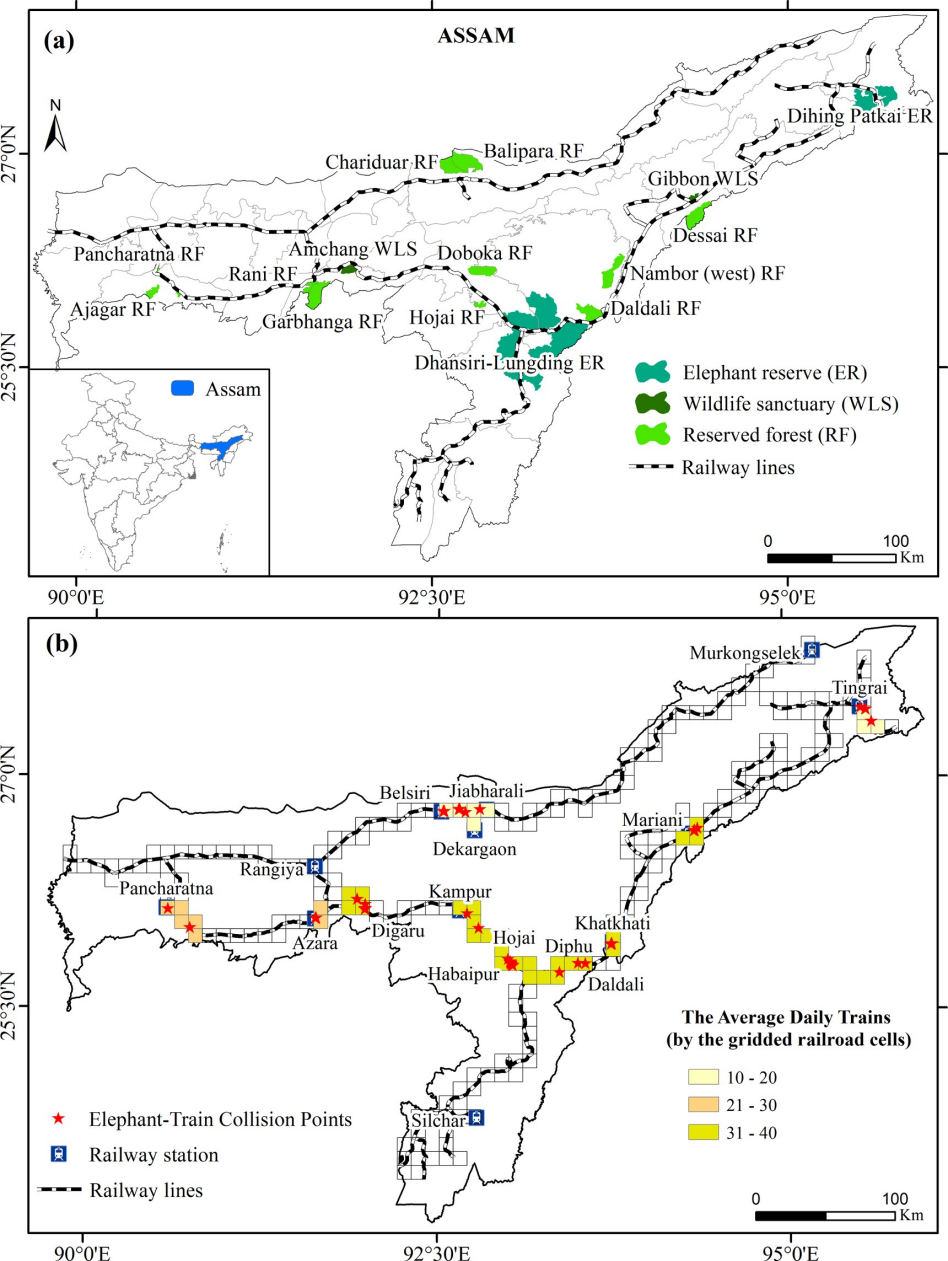
The study area is depicted with the location of Assam in India (inset, a). The important elephant reserves, wildlife sanctuaries and reserved forests are shown along with the railway segments (a). The elephant train collision points and the average daily trains per gridded railroad cells are shown in (b).

The present study selected 11 sample railroad segments, which pass through several elephant ranges of Assam, India (Fig 1). A stratified random sampling technique was used to select these segments based on distances from the elephant habitats. Six segments were located near the habitat and were frequently crossed by elephants. The other five railroad segments were relatively far from the habitats and seasonally crossed by elephants. The elephant population along the sample railroads is distributed heterogeneously in the several elephant zones comprising of reserved forests, wildlife sanctuaries and elephant reserves (Table 1). These elephant zones extend over an area of 3568.21 km^2^ where 769 elephants accounted for an estimated density of 0.22 elephant per square km (Table 1). The study area is significant in that it hosts a tenth of the world’s Asian elephant population of some 5700 elephants [59].

**Table 1:** Distribution of elephant population in the adjacent elephant zones of the sample railroads.

Railroads	Adjacent elephant zone	Area of elephant zone (km^2^)	Number of elephants
Tingrai-Powai	Dihing Patkai ER	461.02	124
Mariani-Nakachari	Dessai RF & Gibbon WLS	48.95	35
Khatkhati-Dimapur	Nambor (west) & Diphu RF	303.17	68
Daldali-Dhansiri	Daldali RF	123.32	54
Habaipur-Diphu	Dhansiri-Lungding ER	1375.41	201
Kampur-Hojai	Hojai & Doboka RF	122.84	31
Belsiri-Dekargaon	Chariduar RF	261.07	53
Rangapara-Jiabharali	Balipara RF	189.76	57
Panikhaiti-Digaru	Amchang WLS	78.64	48
Kamakhya-Azara	Rani & Garbhanga RF	551.87	80
Pancharatna-Dudhnoi	Ajagar & Pancharatna RF	52.16	18
Total		3568.21	769

**Note:** ER = elephant reserve; WLS = wildlife sanctuary; and RF = reserved forest

Source: Elephant Census Report, Assam, 2017.

A GIS database of the study area was developed using ArcGIS 10.1 (www.esri.com). The database consists of (a) elephant train collision and casualty data (1990–2018), (b) elephant population data (2017), and (c) railway traffic data (1990–2018). Railway tracks were represented by adjoining 10×10 km grid cells, where all the variables were measured for each cell. This chosen cell size (100 km^2^) represents the minimum range size of Asian elephants [60]. The elephant zones along the sample railroads were conservatively enclosed by the grid cells.

### 2.2 Data collection

**Fig 2:**
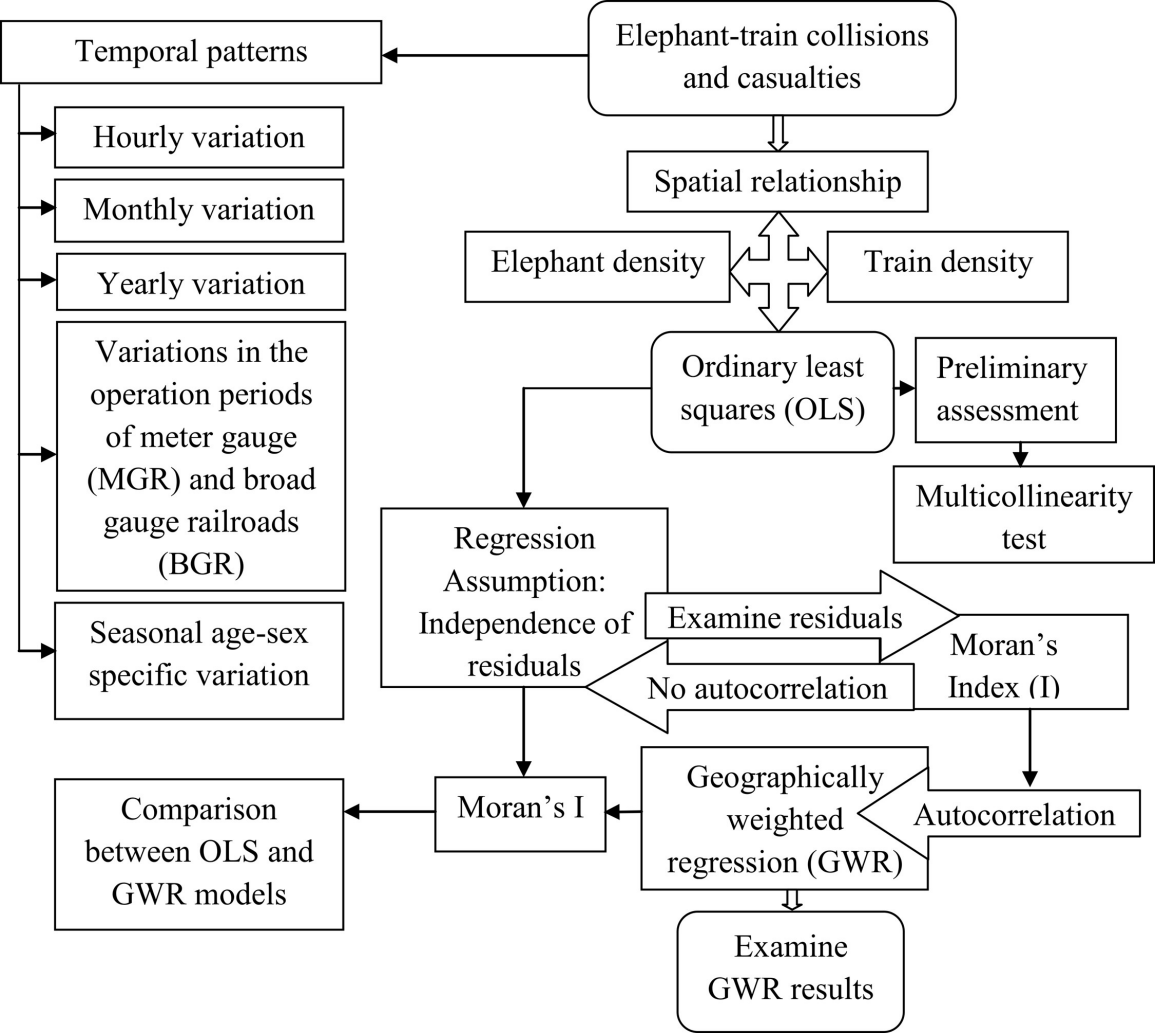
Flowchart showing the methodology adopted to determine spatially varying relationships between elephant-train collision and casualties vis-a-vis elephant and train densities in 10 km^2^ grid cells of 11 railroad segments using ordinary least squares and geographically weighted regression.

To assess the spatio-temporal patterns of ETCs and casualties, the data concerning such incidences in the railroad segments were obtained from the Principal Chief Conservator of Forest (PCCF) and Chief Wildlife Warden, Assam; the divisional forest offices of Digboi, Jorhat, Karbi Anglong (east), Karbi Anglong (west), Nagaon south, Sonitpur west, Kamrup east and Goalpara; and news media sources over the period 1990 to 2018. Such data recorded the location, date and time of collision incidences, number of elephant casualties and their gender and age groups. However, since some location data of ETCs were missing for the years 2016–18, these details were acquired through field visits. This first-hand information included global positioning system (GPS) coordinates being collected using a handheld Garmin GPS MAP 64s device. Monthly precipitation dataduring 1990–2018 was obtained from the Indian open government data platform (https://data.gov.in/), to assess the relationship between ETCs and precipitation. Elephant population data of 2017 for the sampled reserved forests and wildlife sanctuaries was sourced from the respective divisional forest offices of Assam. These data included the size of reserved forests and wildlife sanctuaries, and numerical data pertaining to elephants in and around the former. Moreover, railway traffic data concerning the number, types and speeds of operating trains during the period 1990–2018 was obtained from the northeast frontier railway (NFR) and its divisional headquarters (Tinsukia, Lumding and Rangiya). The overall analysis methods for this study are illustrated in Fig 2.

### 2.3 Temporal analysis

Temporal variations in ETCs and casualties were assessed in the yearly, monthly, hourly and seasonal timeframes. To derive annual rates of ETCs and casualties along the study sites, the total numbers of collision incidences and casualties were divided by the 29 year analysis period (1990–2018). The changing trend of ETCs and casualties over the years was tested by Pearson’s correlation (r) statistics, while t-test was used for monthly variations. The annual number of ETCs and casualties between the operation periods of MGR and BGR were measured by a Mann-Whitney U test. To assess seasonal patterns of age-sex specific elephant casualties, the seasons were categorised into winter (January-February), pre-monsoon (March-May), monsoon (June-October) and post-monsoon season (November-December).

### 2.4 Regression analysis

#### 2.4.1 Definition and validation of variables

In this study, ETC rate was the response variable in the regression model and the elephant and train densities were the predictor variables. The ETC rate was explained as the number of collisions per 10 kilometres of the average length of railroad per gridded cell. The ETC rate for each grid cell was estimated using the following equation:

$$\text{C}=\left(10\times {\text{N}}_{\text{C}}\right)/\left(\text{ARL}\times {\text{Y}}_{\text{C}}\right)$$ (1)

where C is the ETC rate; N_C_ is the total number of collisions; ARL is the average length of railroad per gridded cell, which is calculated by dividing the length of the sample railroad by the number of overlapping gridded railroad cells (Table 2); and YC is the number of observation years of ETC data (29 years in the present study, 1990–2018). Train density was explained as the number of trains per 10 km of the average length of railroad per gridded cell. Train density for each grid cell was estimated using the following equation:

$$\text{TD}=\left(10\times {\text{N}}_{\text{T}}\right)/\left(\text{ARL}\times {\text{Y}}_{\text{T}}\right)$$ (2)

Where, TD is the train density; N_T_ is the average daily trains in the sample railroad; and Y_T_ is the number of observation years of railway traffic data (29 years). On the other hand, elephant density was defined as the number of elephants per 10 km^2^ of the nearby elephant zone of the sample railroad segment. Elephant density for each grid cell was estimated using the following equation:

$$\text{ED}=\left(100\times {\text{N}}_{\text{E}}\right)/\left(\text{AEZ}\times \text{ARL}\right)$$ (3)

where, ED is the elephant density; N_E_ is the number of elephants in the elephant zone; and AEZ is the area of the elephant zone.

**Table 2:** Length estimates of the sample railroads and number of ETC points.

Railroads	Railroad length (km)	^1^No. of gridded cells	^2^Average length of railroad	^3^No. of ETC points
Tingrai-Powai	18.62	3	6.21	5
Mariani-Nakachari	10.32	3	3.44	5
Khatkhati-Dimapur	3.69	2	1.85	4
Daldali-Dhansiri	9.0	1	9.00	5
Habaipur-Diphu	58.23	9	6.47	18
Kampur-Hojai	27.34	5	5.47	2
Belsiri-Dekargaon	24.38	3	8.13	9
Rangapara-Jiabharali	8.21	2	4.11	3
Panikhaiti-Digaru	19.81	4	4.95	6
Kamakhya-Azara	12.53	2	6.27	5
Pancharatna-Dudhnoi	23.08	5	4.62	2

**Note:**- (^1^) No. of gridded cells = Number of overlapping gridded railroad cells on the sample railroad; (^2^) Average length of railroad per gridded cell = Length of the sample railroad divided by the number of overlapping gridded railroad cells. (^3^)No. of ETC points = Number of ETC locations per grid cell. Grid cells and ETC points can be seen in Fig 1.

#### 2.4.2 Regression models

Both OLS and GWR analyses were carried out in ArcGIS 10.1. Before running GWR, a preliminary OLS assessment is crucial to identify multicollinearity or the redundant variables from among the predictor variables [61]. The OLS function automatically inspects multicollinearity using the variance inflation factor (VIF), in which the variable with more than 7.5 VIF value is considered to exhibit multicollinearity. The Koenker (BP) statistic in OLS was applied to examine whether the relationship between ETC rate and elephant and train densities was consistent or non-stationary over geographic space.

GWR processes spatial data using a kernel function and bandwidth method [54]. Two kernel functions are involved in GWR: i) fixed kernel and ii) adaptive kernel. The former specifies a fixed distance, while the latter specifies an optimal number of neighbours to define the spatial context. The adaptive kernel was chosen in this study, since the observations were irregularly distributed and it usually produces less extreme coefficients than the fixed kernel [62]. Fifteen neighbours out of a total of 239 gridded railroad cells were selected to estimate the GWR statistic. The kernel extent in GWR is determined either by cross validation (CV) or the AICc bandwidth method. The latter was adopted in this analyses. GWR generated local regression statistics, such as local R2 values and coefficients for the sample railroad cells and enable a visualization of the relationship. The p-values of model coefficients were estimated using Byrne’s adjustment approach [63] to avoid issues of multiplicity.

#### 2.4.3 Model comparisons

The global R^2^ and AIC_c_ values were used in comparison between OLS and GWR model performances. Generally, a model with a lower AIC_c_ and a greater R^2^ is recognized as a better performance model [64]. The global Moran’s Index (I) was used to check the degree of spatial autocorrelation in both the OLS and GWR residuals using the spatial autocorrelation function in ArcGIS 10.1. Moran’s I generally ranges between -1 and 1 where 1 indicates high positive autocorrelation, while -1 is indicative of highly negative autocorrelation, and 0 indicates the absence of autocorrelation.

## 3. Results

### 3.1 Temporal variations of ETCs and casualties

**Fig 3:**
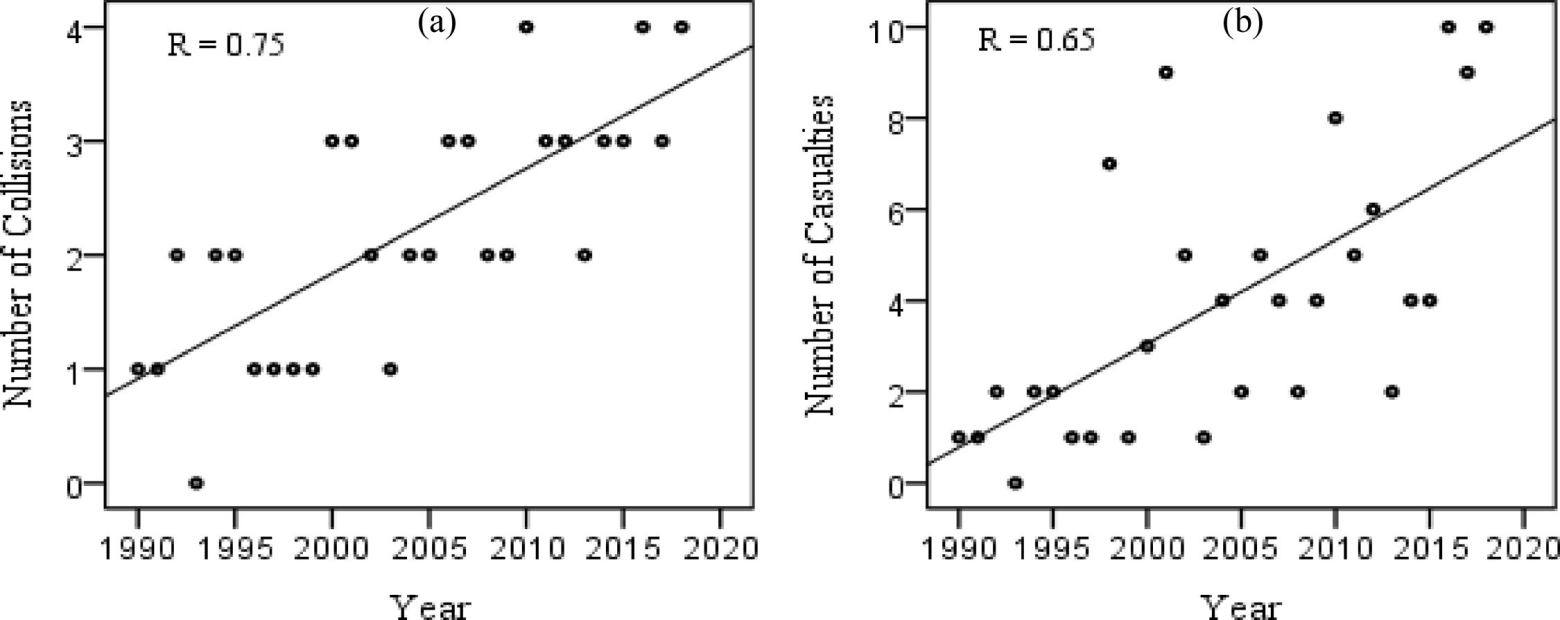
Scatter plots showing increasing trend of (a) elephant-train collisions and (b) casualties over the years.

**Fig 4:**
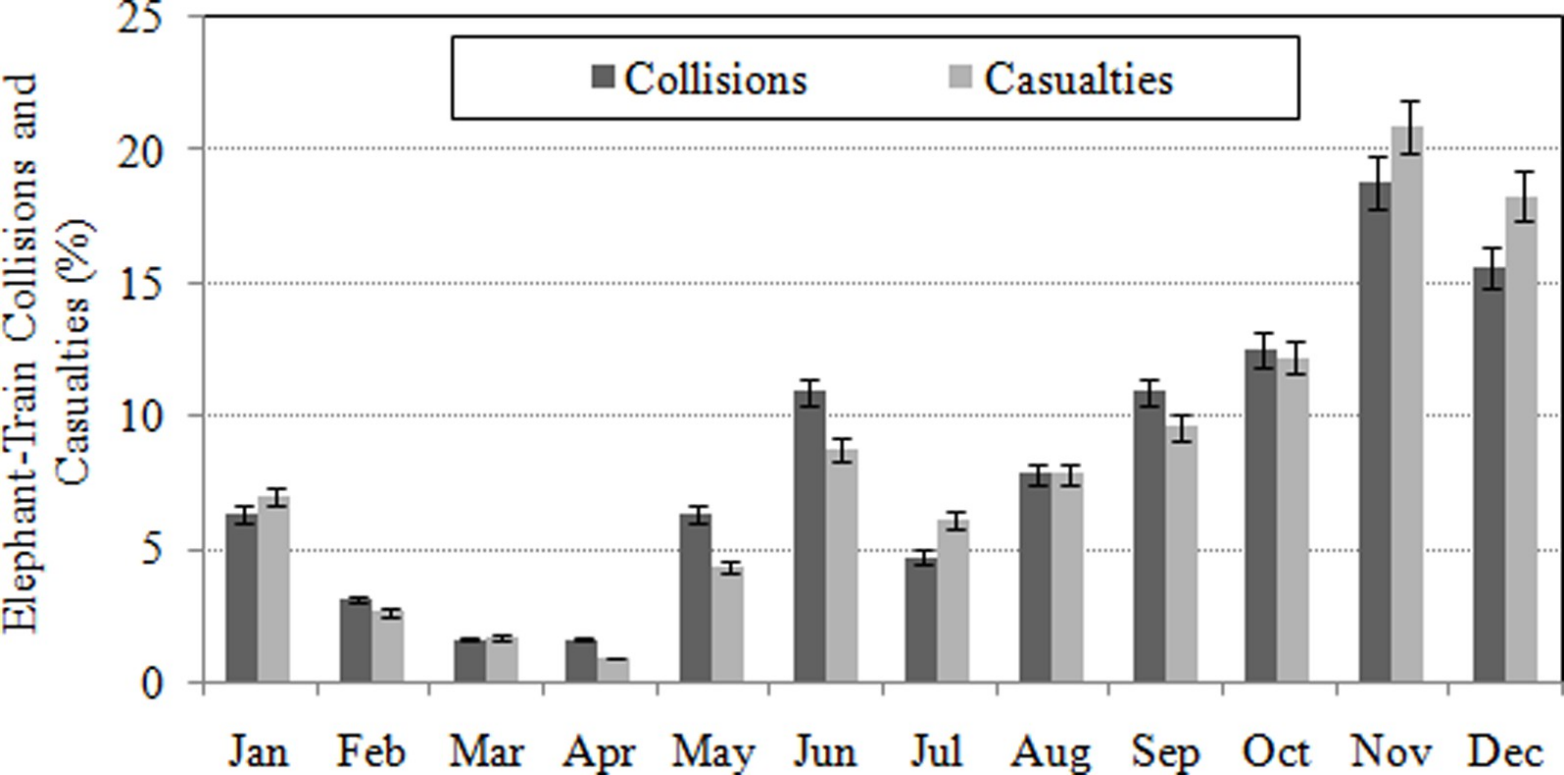
The average monthly rainfall superimposed on monthly variations in the occurrences of elephant-train collisions and casualties (1990–2018).

115 elephant deaths were reported from 64 collision incidences during 1990–2018, with a distinct surge over the years (Fig 3) in the ETCs (Pearson’s r = 0.75, *P*< 0.01) and casualties (r = 0.65, *P*<0.01).With the highest proportions of ETCs (28.1%) and casualties (31.3%), the Habipur-Diphu railroad emerged as a hot spot track of ETCs (Table 3).There was also a significant pattern in the monthly variations of ETCs (t = 5.2, df = 11, P < 0.01) and casualties (t = 4.6, df = 11, P < 0.01). Frequent ETCs and casualties were consistently reported during the months of November and December (Fig 4), wherein the former registered the highest proportions of ETCs (18.8%) and casualties (20.9%). Conversely, the frequency of collisions and casualties were intermittent between the months of January and October (Fig 4). The average monthly precipitation had no significant association with the occurrences of ETCs (Pearson’s r = -0.19, P > 0.05) and casualties (r = -0.30, P > 0.05). However, it was expected that decreasing monthly precipitation would increase ETCs because the frequency of elephant movements tends to have an inverse relationship with precipitation [65].

**Table 3:** Elephant-train collisions and casualties in the sample railroads, 1990–2018.

Railroads	Collisions (%)	Casualties (%)	Casualties/ collision^-1^	Annual rate (Collisions)	Annual rate (Casualties)
Tingrai-Powai	7.8	11.3	2.6	0.18	0.46
Mariani-Nakachari	6.3	5.2	1.5	0.14	0.21
Khatkhati-Dimapur	6.3	4.3	1.3	0.14	0.18
Daldali-Dhansiri	7.8	7.0	1.6	0.18	0.29
Habaipur-Diphu	28.1	31.3	2.0	0.64	1.29
Kampur-Hojai	3.1	6.1	3.5	0.07	0.25
Belsiri-Dekargaon	10.9	7.8	1.3	0.25	0.32
Rangapara-Jiabharali	4.7	7.0	2.7	0.11	0.29
Panikhaiti-Digaru	12.5	10.4	1.5	0.29	0.43
Kamakhya-Azara	9.4	7.8	1.5	0.21	0.32
Pancharatna-Dudhnoi	3.1	1.7	1.0	0.07	0.07
Total	100.0	100.0	1.8	2.29	4.11

**Fig 5:**
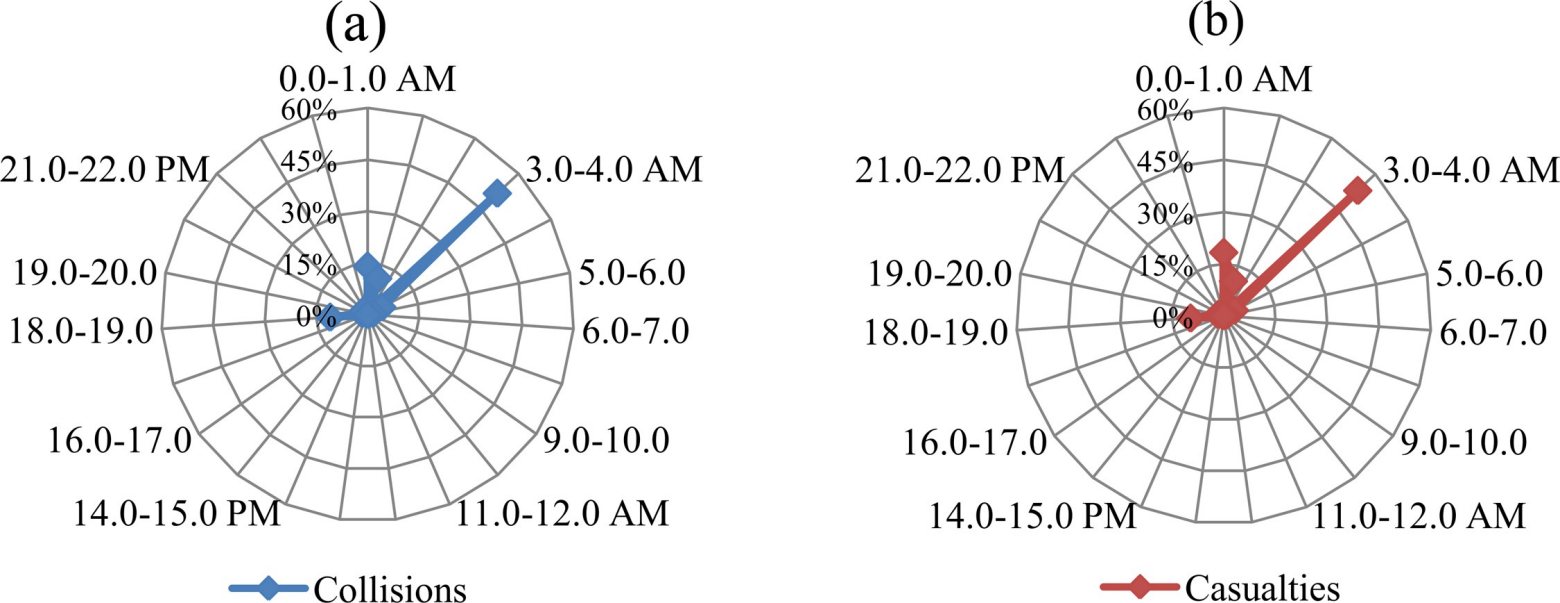
Hourly variations in the occurrences of (a) elephant-train collisions and (b) casualties.

The hourly variations in ETCs and casualties were illustrated using a radar chart (Fig 5). The proportion of ETCs (29.7%) and casualties (33%) climbed markedly in the early morning hours3.00–4.00 a.m., while the midnight hour 00.00–1.00 a.m. also had a noteworthy proportion of ETCs (14.1%) and casualties (17.4%). The proportion of ETCs and casualties in the early morning hours were significantly higher than ETCs (Z = 2.14, P < 0.05) and casualties (Z = 2.16, P < 0.05) occurring towards midnight and in the small hours. No ETCs were reported during the day (6.00 a.m.-6.00 p.m.). This was because elephants being crepuscular in nature, usually prefer to rest in closed canopy forests during the daytime, and tend to forage during the night [9]. Significantly, their foraging activities during the nocturnal hours tend to increase in areas where anthropogenic disturbances in the forested habitats heighten at daytime [66].

ETCs and casualties during the operation of BGR (1998–2018) were significantly higher than those during the operation of MGR (1990–1997). Only 15.6% ETCs and 8.7% casualties occurred during the MGR period, compared to 84.4% and 91.3% percent respectively during the BGR operating (Table 4). Statistically, the two periods displayed significant differences for ETCs (Mann-Whitney U = 27.1, Z = -2.96, P<0.01) as well as casualties (Mann-Whitney U = 16.6, Z = -3.34, P<0.01). BGR facilitates higher traffic volumes and train speeds with heavier loads than those by MGR [9]. Based on the NFR data during the period 1990–2018, the average speeds of trains have substantially increased from 35 kmph in the period of MGR to 60 kmph in the period of BGR in Assam. Railways improve access to natural resources, enable increased migrant influx, and expand market potentials [67], often resulting in large scale land cover change along railroads and their environs through human encroachment, logging, mining, crop-land expansion and spawning road networks [31, 68]. Habitat loss and fragmentation due to land cover change affects the behaviour and movement of Asian elephants significantly [31, 69] since they require large intact forests for their sustenance.

**Table 4:** The proportions of elephant-train collisions and casualties (1990–2018) along the meter and broad gauge railway tracks.

Railroad segment	% Elephant-train collisions		% Elephant casualties	
	Meter gauge railroads	Broad gauge railroads	Meter gauge railroads	Broad gauge railroads
Tingrai-Powai	20.0	80.0	7.7	92.3
Mariani-Nakachari	0	100	0	100
Khatkhati-Dimapur	0	100	0	100
Daldali-Dhansiri	20.0	80.0	12.5	87.5
Habaipur-Diphu	11.2	88.8	5.5	94.5
Kampur-Hojai	0	100	0	100
Belsiri-Dekargaon	57.1	42.9	44.4	55.6
Rangapara-Jiabharali	66.7	33.3	25.0	75.0
Panikhaiti-Digaru	0	100	0	100
Kamakhya-Azara	0	100	0	100
Pancharatna-Dudhnoi	0	100	0	100
Total	15.6	84.4	8.7	91.3

### 3.2 Age-sex specific elephant casualties

Most elephant casualties (51.3%) on the railroads were accounted for from within the adult age group, whereas the proportion of casualties for juveniles (8.7%) and sub-adults (12.3%) were nominal (Table 5). As elephants mature, they experience a shift from mostly natural to human-induced mortality due to an increase in their body masses [70] and movements [71]. Females accounted for most of the casualties (54.8%) in ETCs, in which the proportion of casualties for adult females (34.8%) was relatively higher than those of adult males (16.5%).This difference between male and female elephant casualties was statistically significant (paired *t*-test, *t* = 4.6, df = 10, *P*< 0.01). Increasing female-biased casualties in breeding group could reduce annual reproduction of elephant population [65]. The proportion of calf casualties (26.1%) was also noteworthy possibly on account of their less adroit and swift response to rapid onset dangerous situations. The overall sex ratio of elephant casualties (82.5:100) was nearly representative (Pearson’s r = 0.91, *P*< 0.01) of the living elephant population (81.8:100) while showing no significant difference between those sex ratios (paired *t*-test, *t* = 0.91, df = 10,*P*> 0.05). It appeared that female-biased sex ratio of adult casualties did not significantly affect variation in overall sex ratios between elephant casualties and living population.

**Table 5:** The sex ratios of age specific elephant casualties from train collisions (1990–2018) and living elephant population (2017) in Assam.

Age groups (years)	Elephant casualties			Living elephant population		
	% Male	% Female	^1^Sex ratio	% Male	% Female	^1^Sex ratio
Adults (> 15)	16.5	34.8	47.5	20.4	30.6	66.8
Sub-adults (10–15)	7.8	4.3	180.0	8.6	11.4	74.8
Juveniles (5–9)	5.2	3.5	150.0	7.3	4.7	156.3
Calves (< 5)	15.7	12.2	128.6	8.7	8.3	104.7
Total	45.2	54.8	82.5	45.0	55.0	81.8

**Note:-**^1^Sex ratio = males per 100 females.

**Fig 6:**
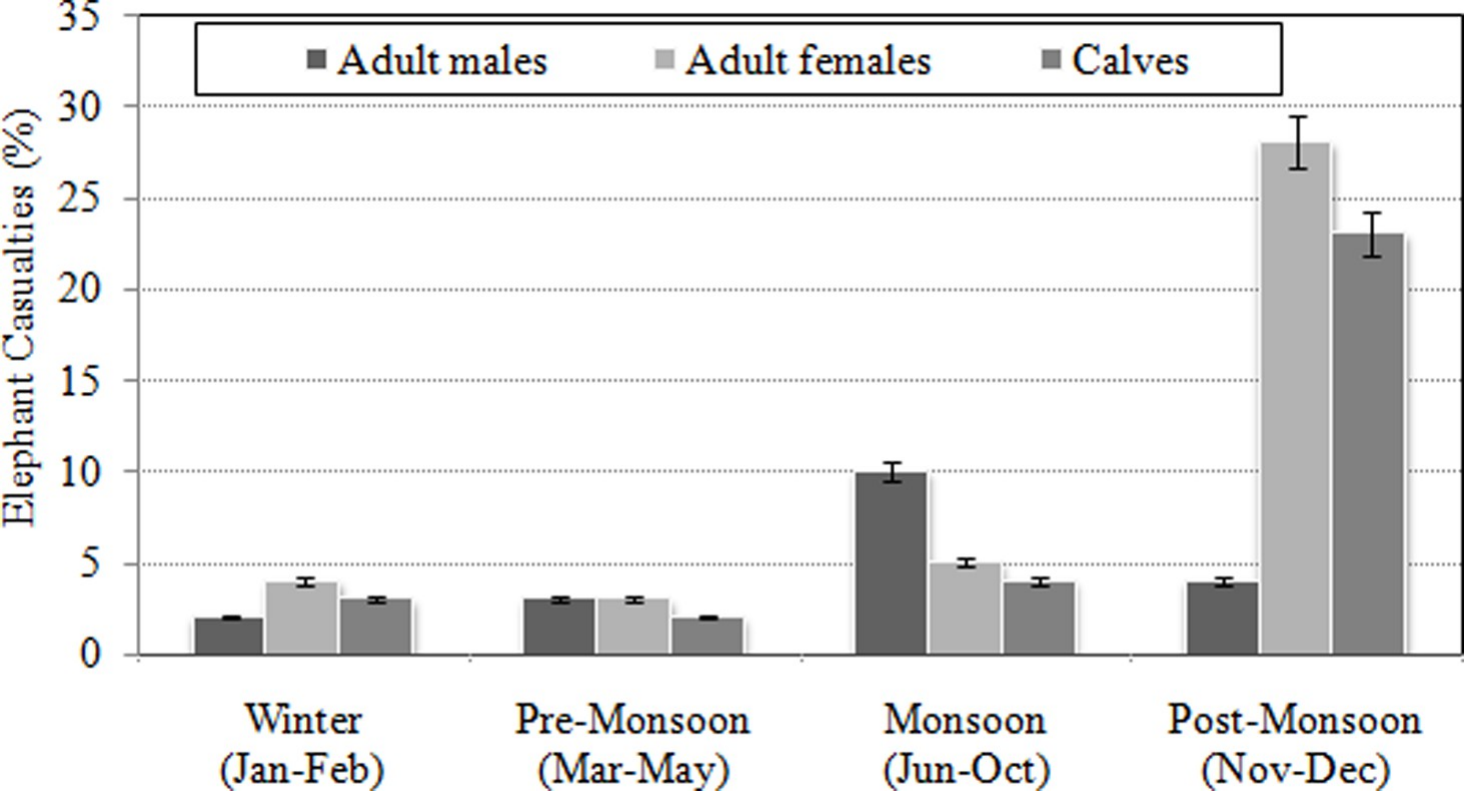
Variation of age and sex specific elephant casualties from train collisions in relation to climatic seasons.

The adult (27.8%) and calf (20.0%) elephant casualties on the railroads were found to be two to fivefold higher (Fig 6) during the post monsoon season (two-way ANOVA, F = 6.12,P< 0.01, Tukey test P< 0.01) compared to those during other seasons (Tukey test P’s > 0.05). More specifically during this season, the percentage of adult female casualties (24.3%) increased substantially (two-way ANOVA, F = 4.2, P< 0.01) with a decrease in adult male casualties (3.5%). A notable proportion of adult male casualties (8.7%) during the monsoon season increased significantly (two-way ANOVA, F = 3.8, P< 0.05) with decreasing adult female casualties (4.3%). The likely reasons for these sex-specific seasonal casualties were, however, unclear though this could the result of sex-specific seasonal migration [72] and differences in behavioural traits including risk taking tendencies and aggressiveness [73].

### 3.3 OLS model versus GWR model

The preliminary assessment of OLS model (Table 6) reveals that the predictor variables do not have any issues of multicollinearity (VIF = 1.78). The significant Koenker (BP) statistics (12.53, P < 0.01) imply that the predictors in OLS do not have a consistent relation to the response variable over the localities. This suggests that the elephant and train densities represent spatial processes that do not act uniformly in the study area.

**Table 6:** OLS regression statistics showing the relative importance of predictor variables measured by the non-spatial coefficient values.

	Coefficient	Standard error	t-statistics	*P*	VIF
Intercept	3.14	0.12	2.8	0.08	
Elephant density	0.09	0.007	3.6	0.001	1.78
Train density	0.16	0.03	3.1	0.001	1.78

Koenker (BP) statistic = 12.53; Koenker (BP) probability = 0.004

**Fig 7:**
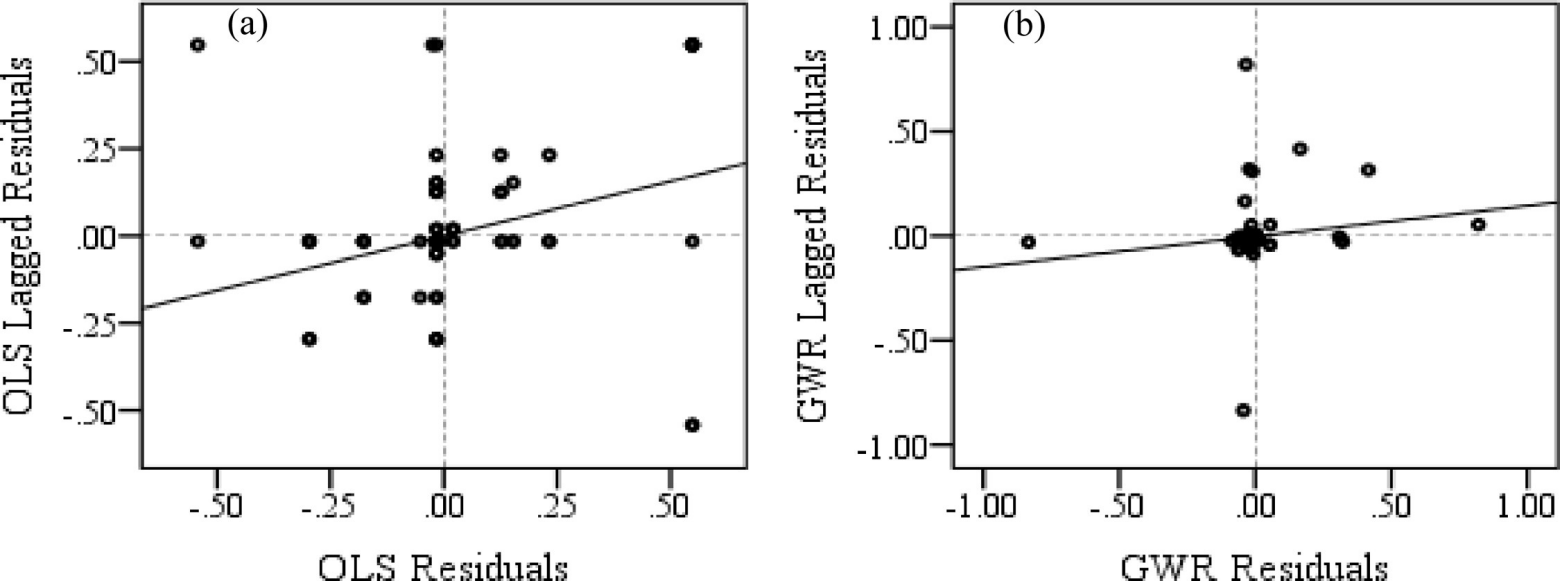
Moran scatter plots for (a) OLS and (b) GWR residuals.

The spatial autocorrelation occurs significantly in the OLS residuals, while a nominal synchrony is discernible in the GWR residuals (Table 7). Such differences are visualized in the Moran scatter plots, where over and under estimated GWR residuals are more evenly distributed compared to the OLS residuals (Fig 7). Moreover, minute aggregations of similar GWR residuals indicate the potential improvement of the model. The R^2^ for the OLS model indicates that both elephant and train densities explain 37% of the variance of ETC rate, which is much lower than the R^2^ for GWR model showing 83% of the variance of ETC rate (Table 8). Moreover, the AIC_c_ estimate of GWR model is significantly lower compared than that of the OLS model (Table 8). Thus, the GWR model provides a better performance model and closer approximation to reality, in the current analysis.

**Table 7:** Comparison between Moran’s I summaries for OLS and GWR models.

Parameters	OLS	GWR
Moran’s Index	0.32	0.06
Expected Index	-0.006	0.000
Variance	0.005	0.000
Z-score	6.26	4.11
P-value	0.002	0.001

**Table 8:** Comparison between the model performances of OLS and GWR.

Parameters	OLS	GWR
Corrected Akaike Information criterion (AIC_c_)	1140.03	1007.46
Coefficient of determination (R^2^)	0.37	0.83

### 3.4 Spatial relationships between ETC rates and the predictors

**Fig 8:**
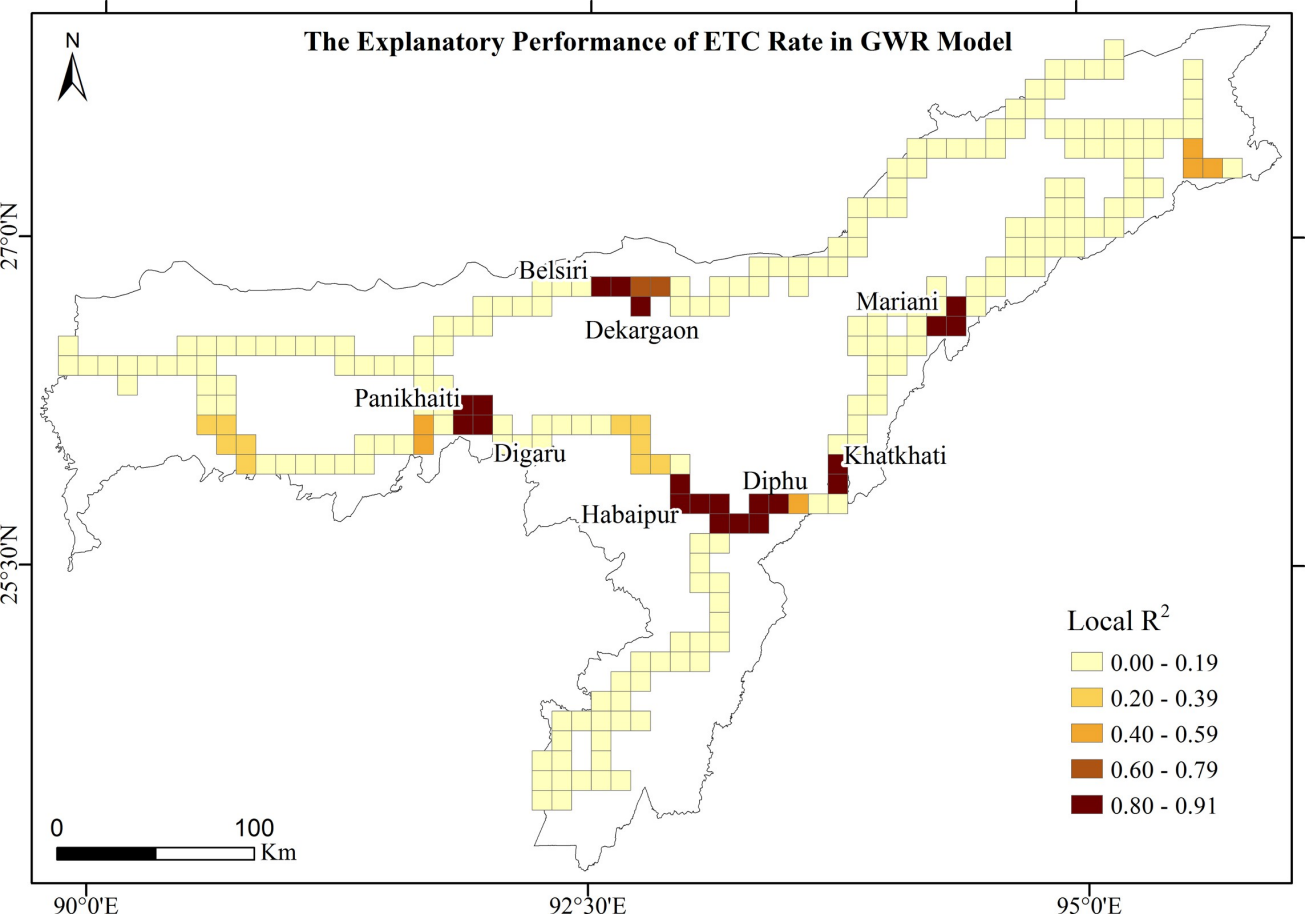
GWR model showing explanatory performances of ETC rates in the prioritized railroads.

**Fig 9:**
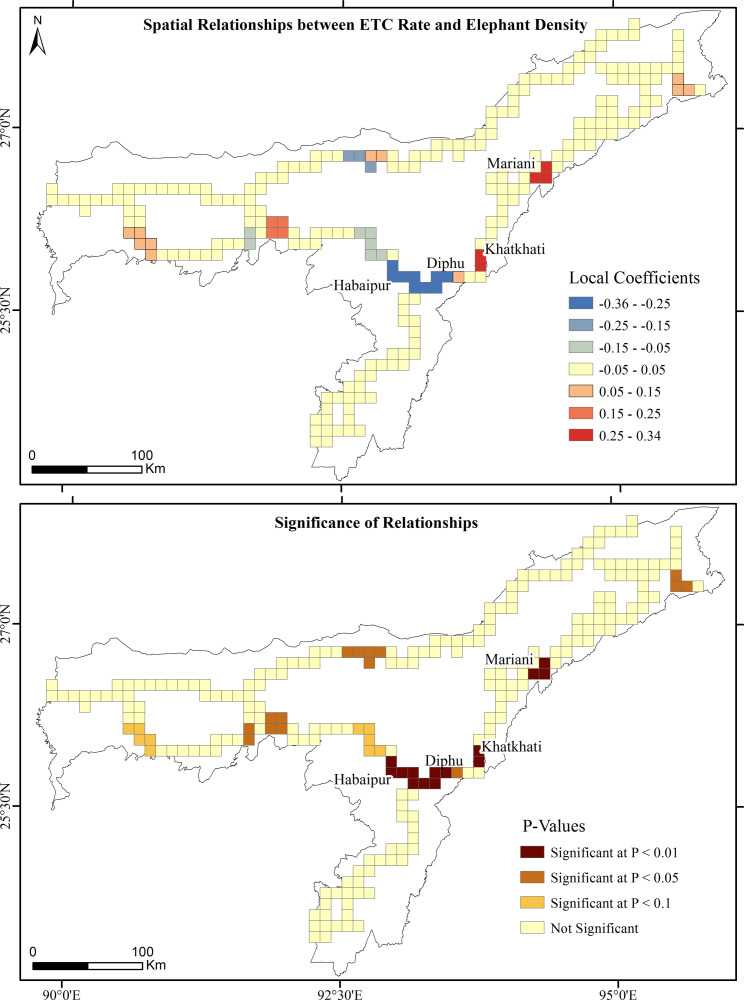
Local coefficients and P-values of GWR model for the spatial relationships between ETC rates and elephant densities.

**Fig 10:**
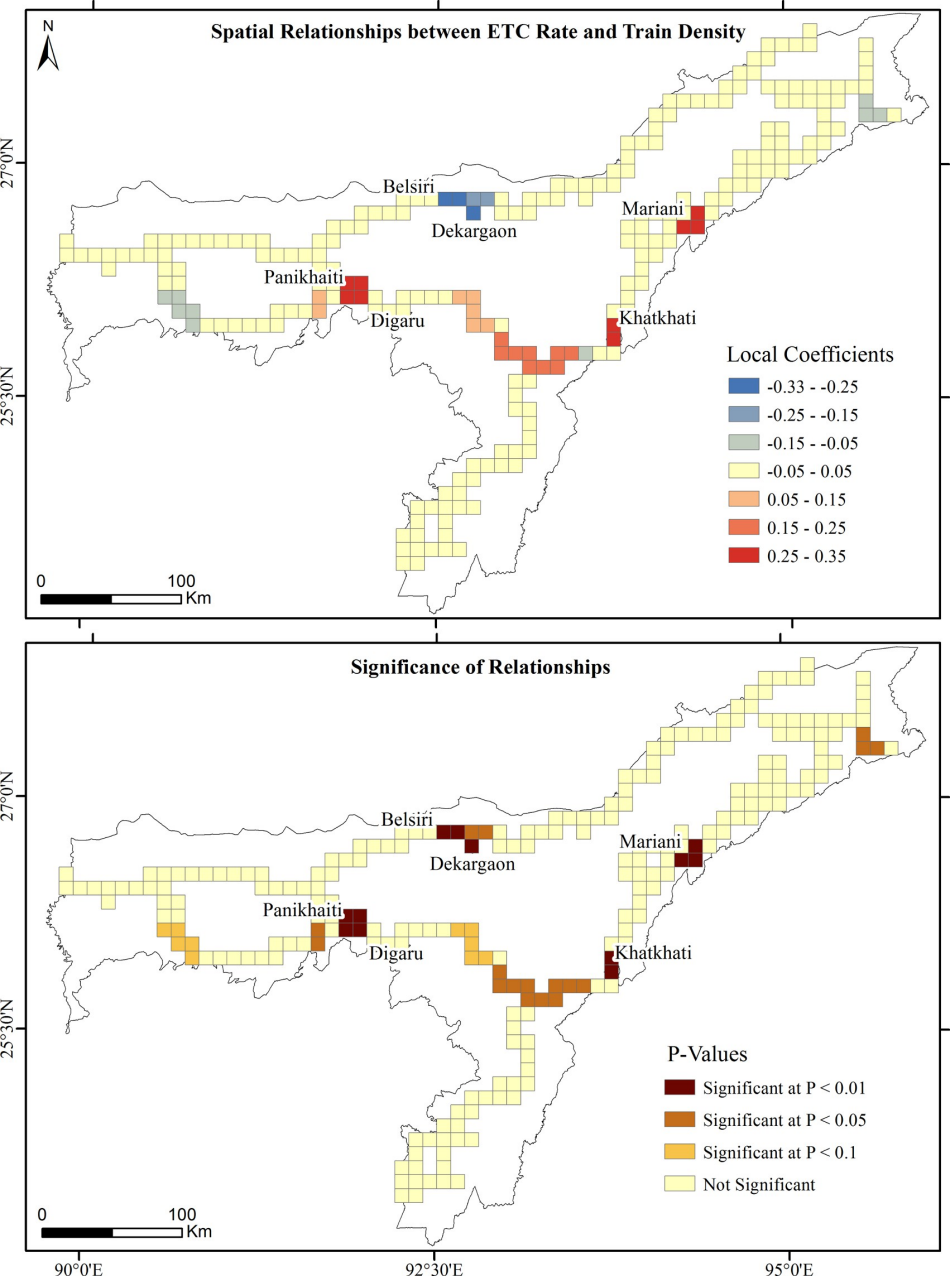
Local coefficients and P-values of GWR model for the spatial relationships between ETC rates and train densities.

GWR estimates the explanatory performance of ETC rates for each grid cell of the prioritized railroads, in which the local R^2^ values range from 0.00 to 0.91 (Fig 8). The higher R^2^ values (> 0.80) are observed in the Mariani-Nakachari, Khatkhati-Dimapur, Habaipur-Diphu, Belsiri-Dekargaon and Panikhaiti-Digaru railroads, where both elephant and train densities explain more than 80% of variations in ETC rates. The local coefficient values estimated by GWR ranged from negative to positive (Figs 9 and 10). At different significance levels, the distribution of coefficients of spatially varying relationships between ETC rates and elephant and train densities depict the necessity of employing local spatial statistics to model such relationships. Both elephant and train densities significantly (P < 0.01) and positively interplay to determine ETC rates in the Mariani-Nakachari and Khatkhati-Dimapur railroads with the estimated coefficients ranging from 0.25 to 0.35 (Figs 9 and 10). For the Habaipur-Diphu railroad, the coefficients of relationships between ETC rates and elephant density were significantly (P < 0.01) negative with values ranging from -0.36 to -0.25 (Fig 9). This intriguing relationship suggests that the elephant population in this region is largely affected by railways through large scale ETCs, where elephants declined alarmingly from 275 in 2005 [74] to 201 in 2017 (Table 1). This also implies that increasing ETCs have a potential to endanger elephant population in their habitats. At significance levels of 0.01 and 0.05, the coefficients of elephant and train densities on ETC rates respectively were significantly positive in the Panikhaiti-Digaru railroad and, conversely, negative in the Belsiri-Dekargaon railroad. These outcomes revealed that the ETC rates also increased significantly with increased elephant and train densities in the Panikhaiti-Digaru railroad and with decreased elephant and train densities in the Belsiri-Dekargaon railroad (Figs 9 and 10). In the latter segment, elephants often crossed and aggregated into large herds during the paddy harvesting season. The Asian elephant is a highly mobile creature capable of moving across a few kilometers to upto 20 kilometers in a day [65] and consequently their density can change temporally over a particular area. On the other hand, even on railway segments with low train densities, high speed trains (>50 kmph) possess the potential to increase casualty rates of elephants.

## 4. Discussion

### 4.1 Temporal patterns of ETCs and casualties

The temporal analysis of ETCs in Assam indicated that accidents, linked with crop raiding and elephant behaviour, spiked at certain hours of the day and months of the year. Following conversion from MGR to BGR faster trains operating also affected ETCs and casualties, that were much higher than those occurring elsewhere in India. All the ETCs during the years 1990–2018 had occurred between 6:00 pm and 6:00 am, demonstrating the importance of frequent elephant crossings at railroads during nocturnal hours. Likewise, November and December were the peak months of ETCs and casualties, a finding that is at variance to those of similar studies in other states of India [9, 75]. In other parts of India elephant mortalities are linked to crop raiding and the maize (May–June) and paddy-harvesting season (Oct–Dec) [9]. In Assam harvesting seasons differ slightly for maize (April-July) and paddy (August-December) [31], but maize is a less important crop compared to the cultivation and acreage of paddy. Thus, the April-July period is comparatively less important in terms of elephant mortality on railway tracks as crop raiding is more of a winter peaking activity. Also, precipitation tends to taper off by October. Thus there were monthly fluctuations in the frequencies of elephant movements in relation to varying vegetation phenology and availability of forage and water [76, 77]. Increasing frequency of annual ETCs over the years was probably due to increasing number and speed of trains, particularly after gauge conversion from MGR to BGR [9]. Gauge conversion led to a twofold rise in train speeds and numbers in India [78]. Generally, a high speed train cannot be halted instantly. Trains running at speeds of 50 km/hour^-1^ need a minimum breaking distance of 600 meters [78]. Therefore, speeding trains even with low traffic volumes were far more perilous to elephants than busy highways [5]. Moreover, there exist seasonal inconsistencies in sex specific casualties, probably due to seasonal climatic conditions [79] and behavioural differences of seasonally migrating individuals [73]. The overall annual casualty rates (4.1 year^-1^) for Assam (Table 3) was nearly five-fold that of previously reported estimates elsewhere in India in the Rajaji National Park, Uttarkhand [8] and north Bengal [9].

### 4.2 Elephant and train density factors affecting ETC rate

The GWR results display heterogeneous relationships between ETC rates and elephant and train densities. The positive relationship between these response and predictor variables in the Mariani-Nakachari, Khatkhati-Dimapur and Panikhaiti-Digaru railroads is mostly consistent with that determined by similar studies elsewhere [23, 80, 81]. Higher density values of elephant population and trains were observed along certain railroad segments (Table 9). The highest elephant density was found along Mariani-Nakachari railroad with an estimated 2.08 elephants per 10 km^2^, followed by Panikhaiti-Digaru (1.23 elephant/10 km^2^) and Khatkhati-Dimapur (1.23 elephant/10 km^2^) railroads (Table 9). A greater presence of elephants along railroads meant a higher susceptibility of risks from approaching trains. Higher train density values existed in the Khatkhati-Dimapur (6.58 trains/10 km) and Mariani-Nakachari (3.14 trains/10 km) railroads (Table 9). Both Mariani and Dimapur are the major nodal points of the NFR and receive substantial impetus from the warehousing, trans-shipment and railway maintenance personnel [82]. Spatially increased train density is often associated with high speed train flows and reduced time interval between two successive trains, during which the presence of narrow and steep railroad embankments can act as death traps for elephants because they cannot elude from those railway corridors quickly enough [83].

**Table 9:** Spatial variation of ETC rates and densities of elephants and trains in the grid cells of the prioritized railroads.

Railroads	ETC rate	Elephant density	Train density
Tingrai-Powai	0.28	0.43	0.95
Mariani-Nakachari	0.50	2.08	3.14
Khatkhati-Dimapur	0.75	1.22	6.58
Daldali-Dhansiri	0.19	0.49	1.27
Habaipur-Diphu	0.96	0.23	1.92
Kampur-Hojai	0.13	0.46	2.17
Belsiri-Dekargaon	0.38	0.25	0.78
Rangapara-Jiabharali	0.25	0.73	1.07
Panikhaiti-Digaru	0.42	1.23	2.68
Kamakhya-Azara	0.28	0.23	1.57
Pancharatna-Dudhnoi	0.15	0.75	1.97

Conversely, ETC rates increased with decreased elephant density in the Habaipur-Diphu railroad. These inconsistent results suggest that decreasing elephant density do not necessarily reduce the frequency of elephant railroad-crossings [84]. Elephants usually follow the migratory paths of their ancestors, notwithstanding the anthropogenic disturbances prevailing in such corridors [85]. There is also a negative relationship between ETC rate and train density in the Belsiri-Dekargaon railroad. Even on railway tracks of low train densities, high speed trains (>50 kmph) showed a tendency to accentuate casualty rates of wildlife in Sweden [81]. Due to lack of technology for monitoring train speeds in India’s elephant landscapes, most trains often tended to operate at speeds well above the maximum permissible limits [7].

### 4.3 Mitigation strategies

The ecological and socio-economic expenses of ETCs and casualties could be enormous, from hazardous train derailments to endangerment of elephants [9, 25]. To minimize such incidences, the NFR and forest departments of Assam have employed several mitigation measures as follows: 1) elephant warning signage, 2) imposition of speed restrictions on trains, 3) clearance of vegetation at railroad verges, 4) installation of auditory deterrent devices, 5) patrolling along railroads, 6) food waste management along the railroads and 7) sensitization programmes for train drivers [86, 87]. Short-term strategies, such as reducing train speed (< 30 kmph), are becoming ineffective [88] with the cutback in transport costs and the increase in the demand for high speed railways in India. Hence, there is an urgent need to address the long-term mitigation strategies so that elephants can be conserved by providing safe passages and survival resources along railway lines. However, the estimated cost of long-term strategies to mitigate ETCs is financially challenging [83] and, therefore, such strategies need to be prioritized on railroad segments posing the gravest risks to elephants. Since ETCs are under likely underestimated and not all ETCs reported by train drivers, a more detailed and robust system of recording of ETCs is needed. Ways and means of using new technological tools and sensors for improved monitoring of these accidents are urgently needed. The delineated hotspots of ETCs and casualties and spatial relationships between ETC rate and predictors in this study can help prioritize when and where ameliorative strategies are needed to minimize collisions and make railways a less deadly threat to elephants. Long-term strategies, such as overpasses or underpasses, need to be accommodated, particularly in the Habaipur-Diphu, Mariani-Nakachari, Khatkhati-Dimapur and Panikhaiti-Digaru railroad segments.

## 5. Conclusions

The present study sought to provide a comprehensive understanding of the temporal pattern and spatial process of ETCs and casualties along railroad segments in Assam. However, there is a probable underestimation of ETCs in data due to restricted access of railway right of way, where only trains’ operators are aware of the majority of ETCs [47]. Not infrequently train-induced elephant casualty data officially recorded only cases reported by the trains’ drivers [89]. Such underestimation might result in data redundancy and imprecise hot-spots of ETCs. However, the present study utilized various sources of information on ETCs, including forest official records, news reports and field based data. Although ETC rates show microlevel spatial relationships with elephant and train densities using GWR model, incorporating monitoring data of elephant movements will certainly improve the accuracy of such assessments. However, direct monitoring of crepuscular and nocturnal elephant movements along the prioritized railroads was practically infeasible due to topographic constraints, absence of lateral roads and lower accessibility along the railroads. Nevertheless, ETCs are an unfortunate fallout of expanding anthropogenic infrastructure slicing into animal habitats that needs to be addressed globally and nowhere more so than in the biodiversity rich Indian province of Assam.
